# Nanocomposites with p- and n-Type Conductivity Controlled by Type and Content of Nanotubes in Thermosets for Thermoelectric Applications

**DOI:** 10.3390/nano10061144

**Published:** 2020-06-10

**Authors:** Katharina Kröning, Beate Krause, Petra Pötschke, Bodo Fiedler

**Affiliations:** 1 Institute of Polymers and Composites, Hamburg University of Technology, 21073 Hamburg, Germany; fiedler@tuhh.de; 2 Leibniz-Institut für Polymerforschung Dresden e.V. (IPF), 01069 Dresden, Germany; krause-beate@ipfdd.de (B.K.); poe@ipfdd.de (P.P.)

**Keywords:** epoxy, carbon nanotubes, thermoelectric, Seebeck coefficient, power factor

## Abstract

In this work, composites based on epoxy resin and various carbon nanotubes (CNTs) were studied regarding their thermoelectric properties. The epoxy composites were prepared by infiltration of preformed CNT buckypapers. The influence of different types of CNTs on the Seebeck coefficient was investigated, namely lab-made and commercially available multi walled carbon nanotubes (MWCNTs), lab-made nitrogen doped MWCNTs (N-MWCNT) and commercially available single walled carbon nanotubes (SWCNTs). It was found that only by varying the lab-made MWCNT content could both n- and p-type composites be produced with Seebeck coefficients between −9.5 and 3.1 µV/K. The incorporation of N-MWCNTs resulted in negative Seebeck coefficients of −11.4 to −17.4 µV/K. Thus, the Seebeck coefficient of pure SWCNT changed from 37.4 to −25.5 µV/K in the epoxy/1 wt. % SWCNT composite. A possible explanation for the shift in the Seebeck coefficient is the change of the CNTs Fermi level depending on the number of epoxy molecules on the CNT surface.

## 1. Introduction

The thermoelectric (TE) effect, discovered in 1821 by Thomas Johann Seebeck, describes the direct conversion of temperature differences into electrical voltage and vice versa via a thermocouple. Thermoelectric devices (TED) create a voltage when there is a different temperature on each side of the constituent materials. Thereby, the applied temperature gradient causes charge carriers in the material to diffuse from the hot side to the cold side. This effect can be used to generate electricity or measure temperature.

The Seebeck coefficient (*S*), defined as the ratio of the generated thermoelectric voltage and the temperature gradient, is a material constant and can be positive or negative, depending on the charge carriers (holes (p-type) or electrons (n-type)). To judge the usefulness of a material in thermoelectric systems, power factor (*PF*) and figure of merit (*ZT*) can be calculated. The power factor considers the Seebeck coefficient (*S*) and the electrical conductivity (*σ*) and is defined by Equation (1).

(1) $$PF=S^{2}\sigma$$

For the calculation of the *ZT*, not only the power factor but also the measuring temperature (*T*) and the thermal conductivity (*κ*) is taken into account and is defined by Equation (2) [1,2].

(2) $$ZT=S^{2}\sigma T\kappa^{-1}$$

Thermoelectric materials can be classified into two main groups, namely inorganic thermoelectric materials and organic ones [3]. Inorganic materials, by far the most investigated class of thermoelectric materials, include tellurides, selenides, skutterudites, silicon-germanium alloys, half Heusler alloys and clathrates [1]. For applications in the middle temperature range (up to 300 °C), tellurides and especially bismuth telluride (Bi_2_Te_3_) is the most investigated and applied alloy. The rare earth elements needed to produce such alloys are expensive, difficult to generate and process and there are some geopolitical risks for constant supply with such materials. In addition, they have a relatively high weight. Among the organic thermoelectric materials whose use is limited to low temperature applications (below 150–250 °C), the intrinsically conductive polymers (ICP), such as polyaniline, polythiophene and poly-3,4-ethylenedioxythiophene:polystyrene sulfonate (PEDOT:PSS) are increasingly studied [4]. They have the advantage of low weight and good processability by solvent processes into variable shapes. They can be combined with inorganic materials with high conductivity or Seebeck coefficient to tune their electrical conductivity and Seebeck coefficient. However, in most cases solvents are needed and the materials are not stable. McGrail et al. [5] summarized the thermoelectric performance of ICPs. In general, their *PF* and *ZT* values are significantly lower than those of inorganic materials.

Kim et al. [6] produced thin films of PEDOT:PSS with different CNT contents and investigated the thermoelectric properties of the composites. They achieved the highest values with a filler content of 35 wt. % SWCNTs. Here the power factor was around 25 µW/mK^2^ and the *ZT* value reached ~0.02.

In the last years, conductive polymer composites (CPCs) consisting of nonconductive polymer matrices filled with conductive fillers were developed. Such materials are available, cost-effective, can be produced and processed easily in different shapes and dimensions and are promising for a wide area of low-temperature everyday life applications.

Polymers have a low density and a low thermal conductivity which is favorable for thermoelectric applications. The needed electrical conductive filler network inside the CPCs can be favorably formed by fillers with a high aspect ratio, such as carbon nanotubes (CNTs), which can already change the property of electrical conductivity at very low filler contents (0.1–5 wt. %). However, due to the typical polymer chain wrapping around the fillers, the contact resistances between neighbored fillers are relatively high resulting in electrical conductivity values which are far below the values of inorganic materials or ICPs. This leads to comparatively low *PF* and *ZT* values, even lower than in ICPs.

Within CPCs, mainly thermoplastic matrices are currently considered, e.g., poly(vinylidene fluoride) (PVDF) [7], polypropylene (PP) [8,9,10,11,12,13,14] and polycarbonate [15,16,17]. Other polymers such as rubber, cellulose [18] or thermoset matrices (epoxy systems) have been studied less, or not at all. Results for different thermoplastic polymers show that the Seebeck coefficient of CPCs can be positive or negative, depending on the polymer matrix type and CNT type. Whereas multi walled CNTs (MWCNTs, namely Nanocyl NC7000 and Applied NanoStructured Solutions LLC CNS-PEG) always lead to p-type behavior, single walled CNTs (SWCNTs, namely OCSiAl Tuball 75% and Unidym HiPco super pure) resulted in certain polymer types, such as different polyamides (PAs) and acrylo butadiene styrene (ABS), in negative Seebeck coefficient values [12]. On the other hand, nitrogen doped MWCNTs always resulted in negative Seebeck values of their composites [12,13]. In these investigations on melt-mixed composites, performed with filler contents between 0.1 and 7.5 wt. %, the maximum positive Seebeck coefficient was 66 μV/K for polybutylene terephthalate (PBT) with 5 wt. % SWCNT and the maximum negative Seebeck coefficient −51.5 µV/K (ABS with 0.5 wt. % SWCNT) [11]. Interestingly, also with carbon nanofibers (CNFs) negative Seebeck coefficients of about −9 µV/K were found when 1–5 wt. % CNF were melt-mixed in polypropylene (PP) [19]. Another way to induce negative Seebeck coefficients is the addition of polyethylene glycol (PEG), certain ionic liquids or non-ionic surfactants (such as polyoxyethylene 20 cetyl ether) to composites which showed positive Seebeck values before this addition. This was shown by Luo et al. [8,9,10,11] in the example of PP with SWCNTs. Another concept to achieve TE materials with high power factors is the use of multi-layered thin-films systems, as shown by Cho et al. [20] by combining polyethyleneimine (PEI)/double-walled CNT films with polyvinylpyrrolidone (PVP)/graphene films in a water-based layer-by-layer (LbL) assembly technique. An 80-bilayer film exhibited a very high conductivity of 300 S/cm and a negative Seebeck coefficient of −80 µV/K air-stable over 60 days with no protection, resulting in a comparatively high power factor at room temperature of 190 µW/mK^2^. Based on the developed materials of PP with SWCNTs, Luo et al. presented a demonstration of a TED with 49 leg pairs which produced a voltage of 110 mV at a temperature difference of 70 K [9].

**Figure 1:**
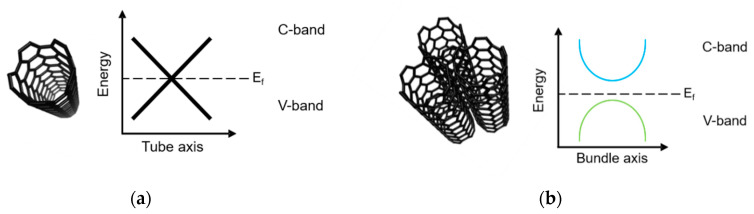
Energy band structures for an isolated SWCNT in vacuum (**a**) and a bundle of SWCNTs in vacuum (**b**) (according to [24]).

Carbon nanotubes along the tube axis are either metallic or semiconducting. Depending on the chirality of the nanotubes, they may be metallic, quasi-metallic with a very small band gap or semiconducting [21]. A couple of scientific questions, especially with regard to the role of the interactions between CNTs and the polymer matrix, are not completely answered until now. In the literature there exists theoretical models to describe the interaction between CNTs with each other and CNTs combined with epoxy matrix monomers based on bisphenol-A. The published results are based on various simulation models [22,23,24]. A number of models were used to discuss the results in this work; the models are described below. Torres et al. [24] describes the interactions between SWCNTs in their model. They suppose that the systems are in vacuum. In the case of a single CNT, the energy bands overlap (Figure 1a). As soon as CNTs are at a certain distance from each other, or agglomerate, this leads to an influence on the energy bands (Figure 1b). The energy bands no longer overlap, a band gap is formed around the Fermi level. In this case, the valence band (V-band) and the conduction band (C-band) no longer overlap. This theoretical model is based on assumptions that it is under vacuum conditions and cannot be directly applied on composites systems based on epoxy-matrix and CNTs.

**Figure 2:**
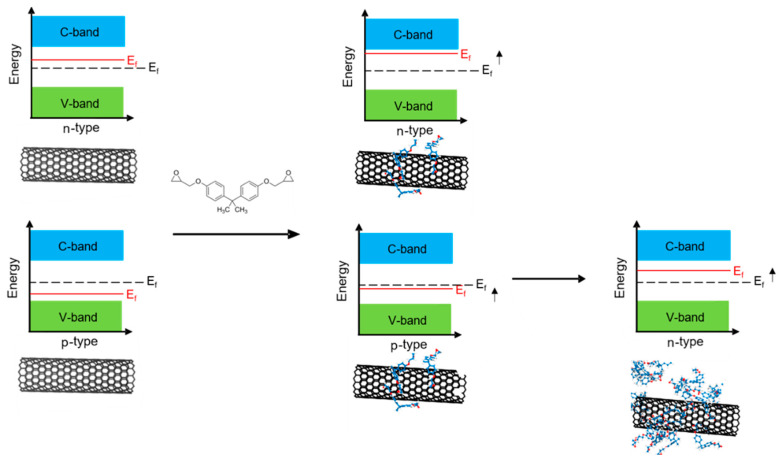
Scheme of possible influence of monomers (here shown for bisphenol-A) on the Fermi level E_f_ of CNTs (according to [22]). C-band is the conductor band, V-band is the valence band. The red line marks the Fermi energy E_f_ of the materials compared to original energy level in the middle between C- and V-band.

The Fermi energy E_f_ is located approximately in the middle of the band gap between the V- and C-band. The Fermi level is the highest energy an electron can have in the ground state of a many-body fermionic system [23]. By doping, the Fermi energy can be shifted in a semiconductor. A p-doping shifts the Fermi energy towards the valence band due to the increased number of positive charge carriers (holes). If the Fermi level of the material shifts nearer to the C-band due to the increased number of negative charge carriers (de-localized electrons), then an n-type material is obtained which has a negative Seebeck coefficient. Thus, the Fermi energy has an important influence on the electrical as well as thermoelectrical properties of a semiconductor. A simulative study by Ghorbanzadeh et al. [22] shows that the Fermi level of SWCNTs can be influenced by the addition of epoxy monomers based on bisphenol-A. The epoxy monomers can bind on the surface of the nanotubes via π–π-stacking. Thanks to the aromatic structure of the resin system, delocalized π-electrons are available to change the Fermi level. The additional electrons can fill positive hole charges of the valence band as well as free spots in the conduction band and thus raise the Fermi level (Figure 2). The Seebeck coefficient is thus reduced and the n-type character of the material increases. The study shows that when more monomers are bound to the surface of CNTs, the Fermi level increases, while the Seebeck coefficient consequently decreases. The influence of cross-linking of bisphenol-A monomers with amine hardeners, typically needed to get final epoxy systems, is not discussed in that study. The results of the simulation work suggest that the Seebeck coefficient decreases further with increasing CNT content in the composite, since effectively more CNT surface area is available for binding the abundant monomers.

Other simulation studies, in which the influence of different monomer types on interactions with SWCNTs was investigated, also show the coating of polymers around the surface of carbon nanotubes [25]. The authors simulated the Fermi level for separated SWCNTs, and considered the number of various monomers, π-stacked on the surface of SWCNTs. In the presence of perfectly separated SWCNTs, different polymer chains get disentangled and align endwise with the SWCNTs to wrap around the nanotube surface.

In the present work composite systems of an epoxy resin based on bisphenol A, an amine hardener and various CNTs were prepared. The effect of the addition of lab-made and commercial CNTs, such as MWCNTs, N-doped MWCNTs and SWCNTs on the electrical conductivity, the Seebeck coefficient, and the power factor was studied. The focus of this work is the investigation of the p- or n-type behavior of the composites.

## 2. Materials and Methods

### 2.1. Materials Used in Composites

Besides MWCNTs synthesized in our lab, for comparison, commercially acquired undoped MWCNTs (NANOCYL NC7000, Nanocyl S.A., Sambreville, Belgium) and SWCNTs (TUBALL75), OCSiAl Europe, Leudelange, Luxembourg) were used without any cleaning steps. For the preparation of CNT containing composites epoxy resin (Hexion, EPIKOTE Resin MGS RIMR 135, Iserlohn, Germany) and resin harder (Hexion, EPIKURE Curing Agent MGS RIMH 137, Iserlohn, Germany) were used. The bisphenol-A based epoxy resin has a low viscosity of 700–1000 mPa·s. The amine hardener is based on 3-aminomethyl-3,5,5-trimethylcyclohexamine.

### 2.2. Synthesis of Undoped and Nitrogen Doped MWCNTs

**Figure 3:**
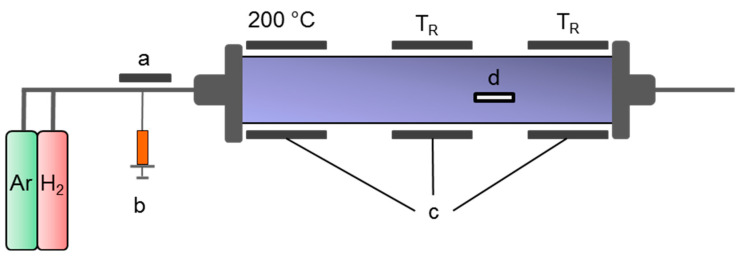
Scheme of the CVD process used: (**a**) evaporation area, (**b**) precursor injection, (**c**) heating zones, (**d**) wafer with CNTs.

Undoped (lab-made MWCNT) and nitrogen doped multi walled carbon nanotubes with three different nitrogen contents (lab made N-MWCNT) were synthesized by the catalytic chemical vapor deposition (CVD) process as described before [26]. A quartz glass tube HSQ 300 (Aachener Quarzglas Technologie Heinrich, Germany, Ø110 mm × 4 mm × 1100 mm) in a horizontal tube furnace HZS (Carbolite Gero, Neuhausen, Germany) with three heating zones (Figure 3c) was used as CVD reactor. A silicon wafer (Si-Mat Silicon Materials, N/Phos., Ø100 mm, Kaufering, Germany) (Figure 3d) was placed between heating zone 2 and 3 before heating up to reaction temperature. The reactor was heated up under constant argon (Westfalen, 99.998%, Cottbus, Germany) gas flow (400 mL/min) till the reaction temperature TR (760–960 °C) was reached. The evaporation area was heated up to 200 °C. Hydrogen (Westfalen, 99.999%, Cottbus Germany) was added as reaction gas and argon gas flow was reduced. Constant gas flows between 110–330 mL/min (10:1 v/v) has been used for CNT synthesis. The synthesis started after adding a reaction feedstock (Figure 3b) in the evaporation area (Figure 3a) with constant injection rates of 5.5 mL/h in case of undoped MWCNTs and 5.5–10.5 mL/h for nitrogen doped MWCNTs. The reaction feedstock consisted of ferrocene (Merck, 98%, Darmstadt, Germany) (5 wt. %) and toluene (Alfa Aesar, 99.5%, Kandel, Germany) for undoped MWCNTs. For doped MWCNTs, pyridine (Roth, ≥99%, Karlsruhe, Germany) (5–95 wt. %) has been added to the feedstock. The variation of nitrogen in the synthesized MWCNTs was achieved by Plunket et al. [26]. After adding 27.5 mL precursor solution the injection was stopped and thus the synthesis was terminated. The reactor was cooled down under constant argon gas flow. After reaching room temperature the silicon wafer (Figure 3d) was replaced and the MWCNTs were removed from the substrate by a razor blade.

### 2.3. Preparation of Buckypapers

**Figure 4:**
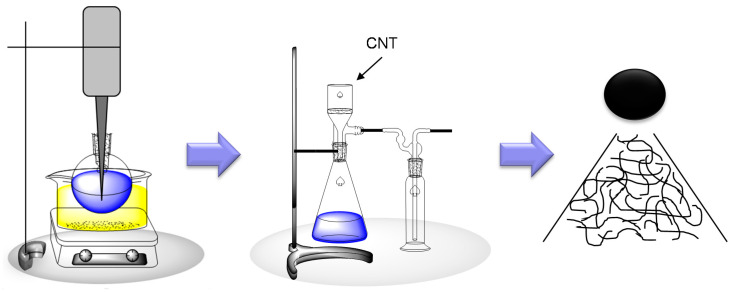
Scheme of CNT buckypaper preparation; tip-sonication (**left**), filtration under lower pressure (**middle**), resulting buckypaper (**right**).

**Figure A1:**
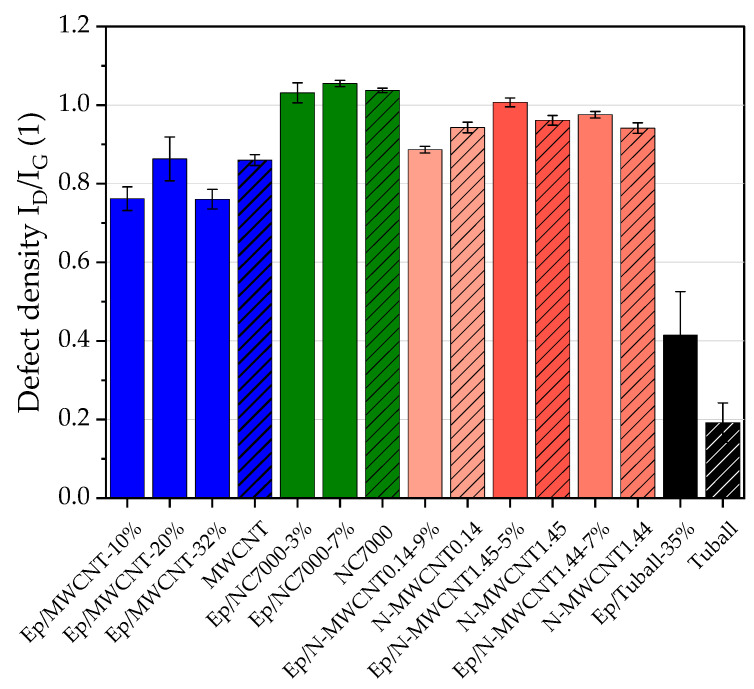
Defect density expressed as the ratio of *I_D_*/*I_G_* from RAMAN investigations of in lab-made undoped MWCNTs, commercial undoped MWCNTs, lab-made nitrogen doped MWCNTs, commercial SWCNT and their composites.

MWCNT buckypapers were processed into composites within a maximum of two weeks after their production. The preparation includes three steps (Figure 4). In the first step 100–300 mg CNTs were dispersed in 100 mL methanol (Kraemer & Martin GmbH, Sankt Augustin, Germany) by tip-sonication (amplitude 30%, 30 min, SONOPULS HD 2200, Bandelin, Germany) at room temperature. Afterwards the CNTs were filtrated, by using filter paper (Schleicher & Schuell, 595 round filter Ø55 mm, Dassel, Germany) and perforated captone foil under lower pressure, by using a constant glass drip diameter and then dried at 80 °C overnight. The resulting buckypaper was removed carefully from the filter. The buckypapers had different thicknesses depending on the amount of CNTs. Further processing of the buckypapers was done by a controlled infusion process. Subsequently, the fiber volume content was determined by a wet chemical digestion. Raman spectroscopy of the buckypapers and the corresponding composites verified that the manufacturing process using ultrasonic tip sonication does not damage the different MWCNTs significantly (see Figure A1).

### 2.4. Vacuum Infusion of Buckypapers with Epoxy for Sample Preparation

**Figure 5:**
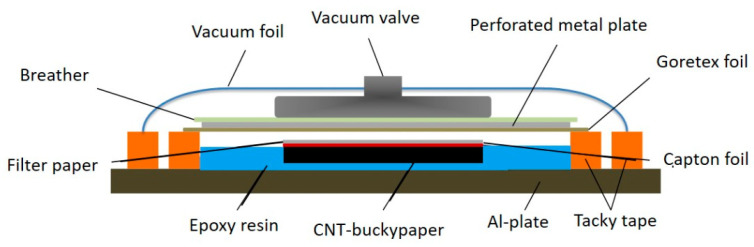
Scheme of the infusion set-up.

The buckypapers were infused with epoxy resin under vacuum in an assembly as shown in Figure 5. The buckypaper, covered with captone foil and filter paper, located on an aluminum plate was placed in a reservoir, limited by tacky tape and filled with ca. 4 mL epoxy resin/hardener mixture. The reservoir was closed with a Gore-Tex foil, a perforated metal plate and a breather. The setup was closed with a vacuum foil. After reaching 80 °C the vacuum infusion started. The infusion time was 2 h. After cooling down to room temperature the composite was removed from the aluminum plate. The resulting filler content in the composites depends on the permeability of the buckypaper during the infusion process.

The infused buckypapers were cut into 10 mm × 30 mm pieces using a saw (ATM-ADVANCED MATERIALOGRAPHY, BRILLANT 200, Mammelzen, Germany). The ends were sharpened with sandpaper and coated with silver paste (Plano GmbH, Wetzlar, Germany) to improve the electrical contact to the contacts of the measurement device of the electrical conductivity (Figure 8b).

**Figure 6:**
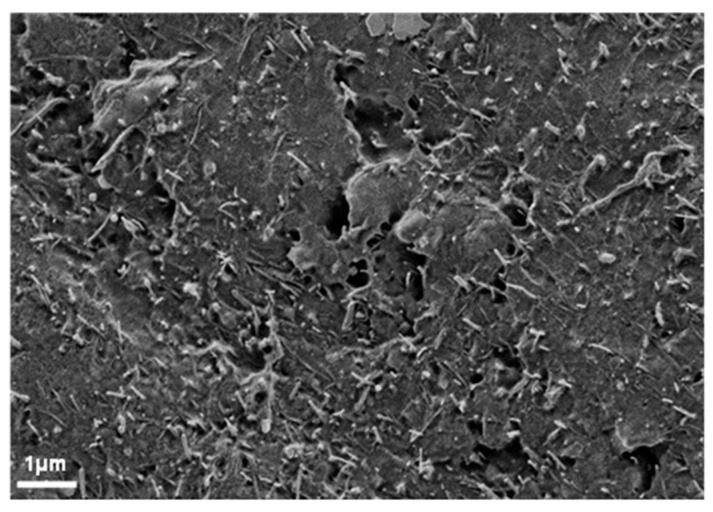
SEM image of the cross section of a composite Ep/MWCNT-10% (representative for all specimens).

To characterize the final sample quality achieved, an SEM image of a cross-section of a composite material is shown in Figure 6. At the surface, some pores can be seen, which might affect the measured values of the electrical conductivity. As such pores have occurred in every production of composites materials, the error can be ignored as it affects all results uniformly.

**Figure 7:**
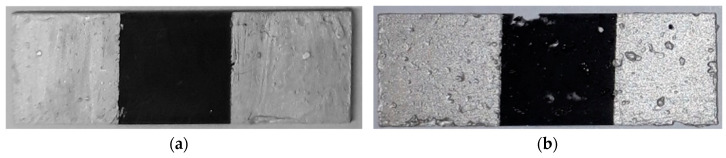
Composite specimens (30 mm × 10 mm × 0.5 mm), partially covered with silver paste; (**a**) Ep/MWCNT-10% (undoped MWCNTs, representative for all undoped specimens) with less and smaller pores; (**b**) nitrogen doped MWCNTs Ep/N-MWCNT1.45-5% (1.45 wt. % N of CNT) with larger and more pores (exemplary for all undoped specimen).

In the case of the N-doped MWCNT materials, the surfaces of the composite in Figure 7b showed a higher porosity, unlike the other samples (Figure 7a). Possibly, the electrical conductivity is influenced by the rough sample surface and the lower conductive area.

The following table (Table 1) shows the nomenclature of the composites including the CNT contents of the samples and the nitrogen contents of the N-MWCNTs.

### 2.5. Characterization

Carbon and nitrogen content of the lab made nitrogen doped CNTs was determined by elemental analysis using a CHNS (Carbon-Hydrogen-Nitrogen-Sulphur) macro analyzer vario MACRI cube (Elementar, Langenselbold, Germany).

Scanning electron microscopy images for samples were acquired using a Supra 55 VP FEG (Carl Zeiss AG, Oberkochen, Germany) scanning electron microscope (SEM) at 5 kV acceleration voltage. The samples had been broken and sputtered with a thin gold film.

The CNT content in the composites was been determined by wet chemical methods. An acid digestion was carried out for this purpose DIN EN 2564.

**Figure 8:**
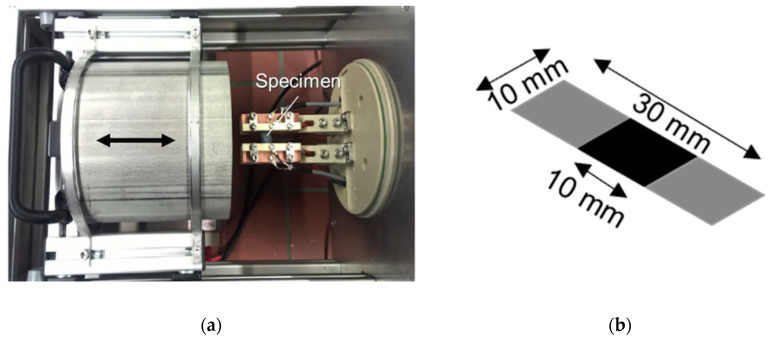
Picture of (**a**) Seebeck measuring device and (**b**) scheme of specimen for thermoelectrically measurements with a thickness of 0.5–0.9 mm (the thickness depends on the manufacturing steps).

The thermoelectric characterization was carried out in a Seebeck measuring device developed at IPF Dresden (Figure 8a). More details are given in following references [12,18,27]. The measuring temperature was set to 313.15 K (40 °C), with a temperature variation range around this temperature of ±8 K in which the slope was used as the measure for the Seebeck coefficient at 40 °C. The measurements of the electrical volume resistivity were done using the same equipment applying the 2-wire technique. The free sample length between the silver coated ends was 10 mm.

## 3. Results and Discussion

### 3.1. Seebeck Coefficient

**Figure 9:**
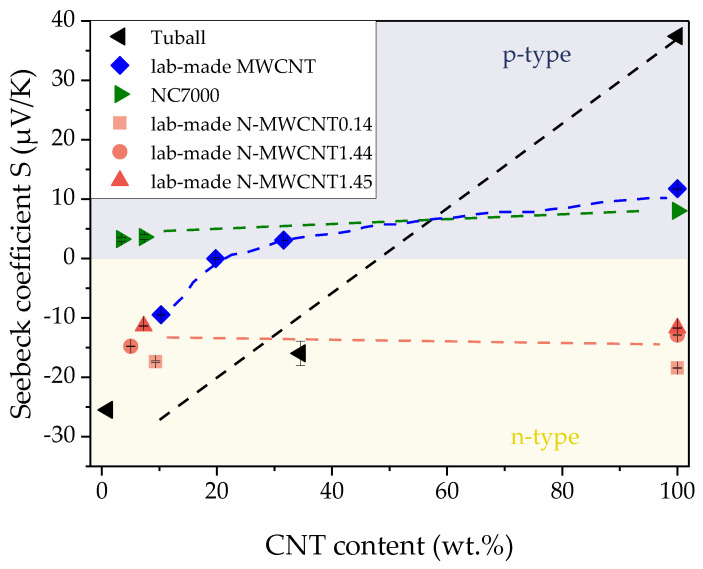
Seebeck coefficient of CNT buckypapers and epoxy composites in dependence on the CNT content sorted by color: lab-made MWCNT (**blue**), commercial MWCNTs (**green**), lab-made N-MWCNTs (**red**), commercial SWCNT (**black**).

The Seebeck coefficient *S* of CNTs and their composites in dependence on the CNT content is shown in Figure 9 and listed in Table 2.

As expected, the *S* values for all buckypapers of undoped CNTs (lab-made MWCNT, Tuball SWCNT, NC7000 MWCNT) were positive indicating a p-type behavior of these materials. The three different kinds of lab-made nitrogen-doped MWCNTs show n-type behavior deduced from negative *S* values. This is in good agreement with other investigations of N-MWCNT reported in Krause et al. [12,13].

The thermoelectric properties of epoxy composites were strongly dependent on the CNT content. The buckypaper from lab-made MWCNTs (blue symbols in Figure 9) had a p-type character with a Seebeck coefficient of 11.8 µV/K. The corresponding epoxy composites with 10.3, 19.8 or 31.6 wt. % MWCNT result in Seebeck coefficients of −9.5, 0.0 and 3.1 µV/K, respectively. It is interesting to note that despite the p-type character of the lab-made MWCNTs, the composites show a significant change in the type of thermoelectric behavior from negative *S* values at low loadings to positive *S* values at higher CNT contents.

Comparable results could be observed for p-type SWCNT and their composites (black symbols in Figure 9). At the low SWCNT content of 1 wt. % in epoxy, an *S* value of −25.5 µV/K was measured. With increasing SWCNT content, the Seebeck coefficient changed to a less negative value (−16 µV/K, 34.5 wt. % SWCNT).

The Seebeck coefficient value for NC7000 MWCNT and their composites was always determined as positive. Whereas NC7000 buckypaper had a *S* value of 8.0 µV/K, the corresponding composites with 3.4 and 7.3 wt. % MWCNTs result in slightly lower Seebeck coefficients of 3.3 and 3.6 µV/K, respectively.

For all composites filled with undoped CNTs, the Seebeck coefficient of the buckypaper was always higher than that of the composites. However, the decrease in the Seebeck coefficient by adding epoxy to the CNTs is much less pronounced for NC7000 than for the lab-made MWCNTs or SWCNTs.

The buckypapers of the N-doped MWCNTs (red symbols in Figure 9) with nitrogen contents of 0.14, 1.44 or 1.45 wt. % resulting from the different synthesis process parameters given in Section 2.1 all have negative Seebeck coefficients (−18.5 µV/K, N-MWCNT0.14; −12.9 µV/K, N-MWCNT1.45; −11.7 µV/K, N-MWCNT1.44). The *S* values of the composites were each in a similar range. In contrast to all doped CNTs, the thermoelectrical properties of the undoped CNTs are hardly changed by the incorporation into the epoxy composites. 

### 3.2. Discussion of the Dependence of the Seebeck Coefficients on CNT Content

Depending on the type of CNT, large changes in the Seebeck coefficient values were observed for CNT content between 1 and 100 wt. %. The Tuball SWCNT material, especially, has already been incorporated into different matrices and can be used for a comparison. In contrast to present work, CNTs contents of only up to 7–10 wt. % were incorporated in different thermoplastic matrices for thermoelectrical applications. However, a comparison between *S* values of composites and buckypapers can be done. For ABS or PA6 composites, the *S* value of Tuball buckypaper (37.4 µV/K) switches to −57.1 µV/K (0.5 wt. % Tuball) or −59.8 µV/K (1 wt. % Tuball) [12]. For Ep/Tuball-1% a Seebeck coefficient of −25.5 µV/K was measured. For ABS composites filled with 2 wt. % Unidym HiPco super pure SWCNTs a Seebeck coefficient of −16.8 µV/K was determined, whereas the HiPco buckypaper showed an *S* value of 28.6 µV/K [12]. 

In epoxy composites, for lab-made MWCNTs, the Seebeck coefficient is most strongly influenced when the filler content is low. A negative Seebeck coefficient was measured at a CNT content of approximately 10 wt. %. At 20 wt. %, the material has a Seebeck coefficient close to zero and therefore exhibits almost no thermoelectric properties. At a CNT content of 30 wt. %, the material then exhibits a positive Seebeck coefficient. In all cases it is remarkable that a negative Seebeck coefficient was determined in composite materials although the CNTs were p-type materials. At this point, however, it should also be noted that for composites with commercial NC7000, negative Seebeck coefficients were obtained neither in thermoplastics [12] nor in epoxy. 

A switching from p-type CNTs to n-type CNT composites, to our best knowledge, has not been reported so far for epoxy/CNT composites. However, Krause et al. [12] related such behavior for some Tuball SWCNT filled thermoplastic composites to the existence of amide or amine groups in the polymer chains (e.g., polyamide (PA), acrylonitrile butadiene styrene (ABS)). It was concluded that the SWCNT doping is connected to the electron-donating effect of these nitrogen containing groups. An amine group is also present in the cured epoxy composite. This group is formed when the amine group of the curing agent reacts with the oxirane group of the epoxy. With regard to the theoretical publication of Ghorbanzadeh et al. [22], matches are seen with the results described here. With increasing epoxy content in the composite (or lower CNT content) the Seebeck coefficient decreased.

Ghorbanzadeh et al. [22] and Yang et al. [25] simulated the Fermi energy for ideal SWCNTs, and considered the number of epoxy monomers, π-stacked on the surface of SWCNTs (Figure 2). The result of the simulation shows that the Fermi level increased with the number of epoxy monomers binding to the surface of the SWCNTs via wrapping or π stacking. The material gets more negative when more monomers are available on the CNT surface (Figure 2). The epoxy matrix based on bisphenol-A can significantly reduce the Seebeck coefficient compared to pure buckypaper. As the filler content increases, the potential surface area of the CNTs increases so that as many monomers as possible can be bound. However, since the CNTs are highly densely packed at high CNT content and it is therefore not possible to occupy the entire surface with monomers, a lower filler content results in a greater change in the Seebeck coefficient.

The negative doping of the CNTs has already raised the Fermi level. Compared to the undoped CNTs, not as many free electrons can be taken up. The Fermi level is approaching saturation. Therefore, the influence of the epoxy matrix on negatively doped MWCNTs is low. That molecules or polymers can act as doping for SWCNTs has been shown by Nonoguchi et al. [28] in his research on SWCNT/dopant dispersions from which buckypapers were made. Based on thin SWCNT films (*S =* 49 µV/K, *σ* = 36 S/cm) it could be shown that Seebeck coefficients between −73 µV/K (triphenylphosphine, *σ* = 49.8 S/cm) and 88 µV/K (carbazole, *σ* = 8.7 S/cm) can be achieved.

### 3.3. Electrical Conductivity of Buckypapers and Composites

**Figure 10:**
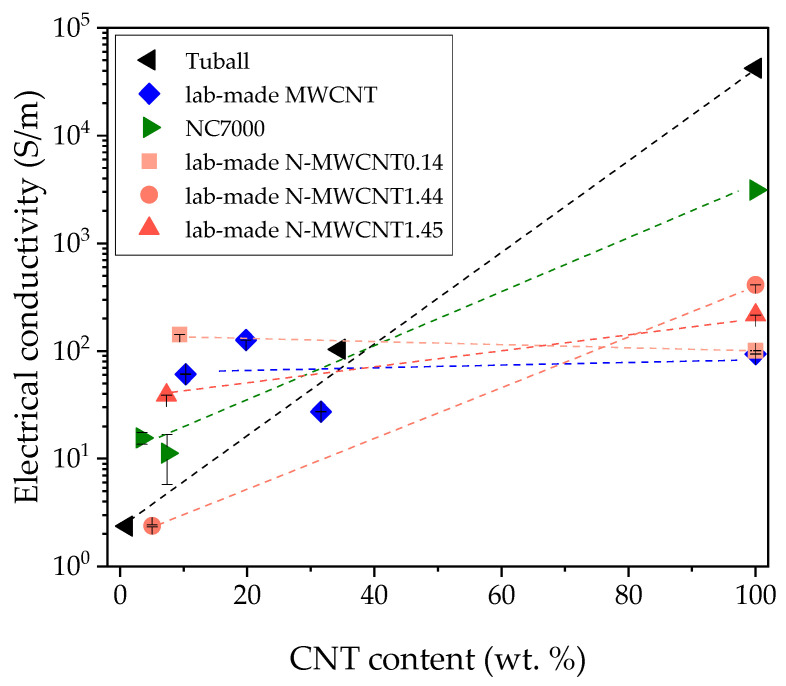
Electrical conductivity of CNT buckypapers and epoxy composites in dependence on the CNT content sorted by color: lab-made MWCNT (**blue**), commercial MWCNTs (**green**), lab-made N-MWCNTs (**red**), commercial SWCNT (**black**).

The neat buckypapers (Figure 10) show electrical volume conductivities in the range between 93 and 42,227 S/m. Interestingly, the conductivity values of buckypaper and composites are in very similar ranges for all lab-made MWCNT and lab-made N-MWCNT types. Among all buckypapers, that of lab-made MWCNTs (blue in Figure 10) shows the lowest electrical conductivity (93 S/m) compared to the other buckypapers. The resulting composites show the lowest influence of the epoxy resin on the electrical conductivity, whereas values of 61 S/m (Ep/MWCNT-10%), 126 S/m (Ep/MWCNT-20%), and 27 S/m (Ep/MWCNT-32%) were achieved. The three lab-made N-MWCNTs and their composites (red in Figure 10) show similar trends of volume conductivity.

For NC7000 and Tuball, the conductivity values of the buckypapers are several times higher than the electrical conductivity of the epoxy composites. The NC7000 buckypaper shows an electrical conductivity of 3125 S/m with a much higher value than the lab-made MWCNTs. After infusion with epoxy resin the electrical conductivity decreases to 15 S/m (Ep/NC7000-3%) and 11 S/m (Ep/NC7000-7%). The SWCNT composites (black in Figure 10) achieved electrical conductivities of 2 S/m (Ep/Tuball-1%) and 103 S/m (Ep/Tuball-35%) whereby the Tuball buckypaper had a conductivity of 42,227 S/m which represents a very strong drop in conductivity due to the epoxy addition.

The decrease in electrical conductivity when forming composites from the buckypapers can be explained by the fact that the isolating epoxy chains wrap around [22] the nanotubes, thus leading to enhanced contact resistances at the crossing points of nanotubes in the buckypaper network. Table 2 lists the electrical conductivities and the corresponding power factors.

### 3.4. Power Factor of Buckypapers and Composites

Due to the small sample geometries and limited material availability, the thermal conductivities of the composites could not be determined. However, for the calculation of the ZT value, the thermal conductivity is necessary. In order to be able to make conclusions about the performance of the nanocomposites, instead the power factor *PF* was calculated. The calculated power factors of all composites are summarized in Table 2. They are in the range of 10^−4^ to 0.26 µW/m·K^2^. Only the composite Ep/MWCNT-20% deviates with a value of 1.3 × 10^−9^ µW/m·K^2^, which is due to the very low Seebeck coefficient which is only slightly above zero (Figure 9). The power factors of the composites are lower compared to those of the buckypapers, due to lower electrical conductivity combined with lower Seebeck coefficients. The highest value of 0.26 µW/m·K^2^ was found for the composite Ep/Tuball-35%. A comparable *PF* value could be observed for thermoplastic CNT composites, whereby the CNT concentration is much lower than the 35 wt. % in epoxy. Luo et al. [9,10] reached *PF* values of 0.1–0.145 µW/m·K^2^ for PP composites filled with 2 wt. % Tuball and 10 wt.% PEG. A significant increase of the *PF* value from 0.12 to 0.26 µW/m·K^2^ was achieved for PP/2 wt. Tuball composites if 2 wt. % imidazolium based ionic liquid was added [8]. Krause et al. [12] reported *PF* values of 0.14 µW/m·K^2^ (ABS/5 Tuball; PA6/5 Tuball) or 0.28 µW/m·K^2^ (PBT/4 Tuball). These power factors are relatively low compared to the common semiconductor materials, as it is expected for composite materials based on insulating polymers. Piao et al. [29] examined a system of SWCNTs/polyvinyl alcohol (PVA)/polyethyleneimine (PEI) and found power factors of around 0.002 to ~0.01 µW/m·K^2^ at a CNT content of 20 wt. %. Nonoguchi et al. [28] investigated SWCNTs with different molecular dopants and found power factors between 0 and ~26 µW/m·K^2^ at 32 °C. Kunadian et al. [30] used different dopants for n- and p-type MWCNTs during the synthesis of the CNTs. The power factor increased with increasing temperature (50–300 K) from ~0 to ~5 µW/m·K^2^.

Thus, the results of the power factor of the SWCNTs and the corresponding composites presented in this work reach similar values like thermoplastic/CNT composites described in the literature. However, the *PF* of epoxy/CNT composites are significantly lower compared to doped SWCNT films. Further fundamental understanding of these nanocomposites is required with regard to the perspective of having low cost energy harvesting materials.

## 4. Summary and Conclusions

In summary, new thermoelectric materials based on epoxy resin and different types of carbon nanotubes have been investigated. Based on the principle of infiltration of preformed CNT buckypapers, CNT type and content were varied and the composites were cured. It was shown that the Seebeck coefficient can be strongly influenced by the filler content of undoped (commercial) SWCNTs as well as lab-made and commercial undoped MWCNTs. In case of undoped lab-made MWCNTs, the Seebeck coefficient of the composites can be both, positive and negative, when using the same epoxy matrix and this behavior is only dependent on the filler content. This work has shown that different sign of the Seebeck coefficient can be achieved only when varying the filler content of undoped MWCNTs in the epoxy matrix. This has the advantage, that the typically used CNT nitrogen doping to achieve negative Seebeck coefficients is not needed. As a result, CNT synthesis process time and costs can be reduced as no doping agents have to be used. In following studies, thermoelectric devices (TED) will be produced from the composites developed here and investigated with regard to their thermoelectric properties. Nevertheless, it will be necessary in the future to work on increasing the *PF* values of polymer-based thermoelectric materials to improve their efficiency. In the following work, it would be also interesting to investigate the oxidation stability of the nitrogen-doped CNTs.

## Figures and Tables

**Table 1:** Nomenclature of the all epoxy composites.

Sample Name	CNT Type	CNT Content (wt. %)	Nitrogen Content of CNTs (wt. %)
Ep/MWCNT-10%	Lab-made MWCNT	10.30	
Ep/MWCNT-20%	Lab-made MWCNT	19.80	
Ep/MWCNT-32%	Lab-made MWCNT	31.60	
MWCNT	Lab-made MWCNT	100.00	
Ep/NC7000-3%	MWCNT NC7000	3.43	
Ep/NC7000-7%	MWCNT NC7000	7.48	
NC7000	MWCNT NC7000	100.00	
Ep/N-MWCNT0.14-9%	Lab-made N-MWCNT0.14	9.35	0.14
N-MWCNT0.14	Lab-made N-MWCNT0.14	100.00	0.14
Ep/N-MWCNT1.45-5%	Lab-made N-MWCNT1.45	5.04	1.45
N-MWCNT1.45	Lab-made N-MWCNT1.45	100.00	1.45
Ep/N-MWCNT1.44-7%	Lab-made N-MWCNT1.44	7.29	1.44
N-MWCNT1.44	Lab-made N-MWCNT1.44	100.00	1.44
Ep/Tuball-1%	SWCNT Tuball	1.00	
Ep/Tuball-35%	SWCNT Tuball	34.50	
Tuball	SWCNT Tuball	100.00	

**Table 2:** Comparison of electrical conductivity *σ*, Seebeck coefficient *S*, and power factor *PF* of epoxy/CNT composites and CNT buckypapers.

Sample Name	Electrical Conductivity *σ* (S/m)	Seebeck Coefficient *S* (µV/K)	Power Factor *PF* (µW/(mK^2^))
Ep/MWCNT-10%	60	−9.5 ± 0.1	0.007
Ep/MWCNT-20%	126	0.0 ± 0.1	1.35 × 10^−^^9^
Ep/MWCNT-32%	27	3.1 ± 0.0	2.62 × 10^−4^
MWCNT-100%	93	11.8 ± 0.1	0.013
Ep/NC7000-3%	15	3.3 ± 0.3	1.66 × 10^−4^
Ep/NC7000-7%	11	3.6 ± 0.4	1.47 × 10^−4^
NC7000 [11]	3125	8.0 ± 0.0	0.202
Ep/N-MWCNT0.14-9%	141	−17.4 ± 0.1	0.043
N-MWCNT0.14	100	−18.4 ± 0.1	0.034
Ep/N-MWCNT1.45-5%	2	−14.8 ± 0.1	5.20 × 10^−4^
N-MWCNT1.45	411	−12.9 ± 0.1	0.068
Ep/N-MWCNT1.44-7%	38	−11.4 ± 0.0	0.005
N-MWCNT1.44	215	−11.7 ± 0.0	0.029
Ep/Tuball-1%	2	−25.5 ± 0.2	0.002
Ep/Tuball-35%	103	−16.0 ± 2.1	0.264
Tuball [12]	42,227	37.4 ± 0.9	59.18

## Appendix A

Raman investigations: Carbon nanotubes can be partially or completely destroyed due to a high energy input by ultrasound during dispersion [31]. This leads to a decrease in the electrical conductivity of the tubes and the later composite. In order to investigate the effects of ultrasonic dispersion on the tubes used in this paper, Raman measurements of the CNTs before dispersion and of the composites were made (Appendix A, Figure A1). Raman spectra on CNTs and composites were acquired using a WITEC TS-150 device (WITEC, Ulm, Germany) equipped with a green laser (532 nm). The defect densities of the corresponding composites were calculated by peak height. No significant changes in the defect densities were measured. The defect densities were determined by means of the peak maxima. Thus, the sonication of the CNT before making the buckypapers has no negative effect on the electrical and thermoelectrical properties of the different materials. In case of the SWCNTs the sonication introduces some defects, but in total the defect densities are much lower compared to all MWCNTs investigated.

## References

[1] Zheng X.F., Liu C.X., Yan Y.Y., Wang Q. (2014). A review of thermoelectric research—Recent developments and potentials for sustainable and renewable energy applications. Renew. Sustain. Energy Rev..

[2] Snyder G.J., Toberer E.S. (2008). Complex thermoelectric materials. Nat. Mater..

[3] Gayner C., Kamal K.K. (2016). Recent advances in thermoelectric materials. Prog. Mater. Sci..

[4] Jianyong O. (2018). Recent Advances of Intrinsically Conductive Polymers. Acta Phys. Chim. Sin..

[5] McGrail B.T., Sehirlioglu A., Pentzer E. (2014). Polymer composites for thermoelectric applications. Angew. Chem. Int. Ed..

[6] Kim D., Kim Y., Choi K., Grunlan J.C., Yu C. (2010). Improved thermoelectric behaviour of nanotube-filled polymer composites with poly(3,4-ethylenedioxythiophene) poly(sterenesulfonate). ASC Nano.

[7] Sun Y., Terakite D., Tseng A.C., Naguib H.E. (2015). Study on the thermoelectric properties of PVDF/MWCNT and PVDF/GNP composite foam. Smart Mater. Struct..

[8] Luo J., Krause B., Pötschke P. (2017). Polymer carbon nanotube composites for thermoelectric applications. AIP Conf. Proc..

[9] Luo J., Cerretti G., Krause B., Zhang L., Otto T., Jenschke W., Ullrich M., Tremel W., Voit B., Pötschke P. (2017). Polypropylene-based melt mixed composites with singlewalled carbon nanotubes for thermoelectric applications: Switching from p-type to n-type by the addition of polyethylene glycol. Polymer.

[10] Pötschke P., Krause B., Luo J. (2019). Melt mixed composites of polypropylene with single walled carbon nanotubes for thermoelectric applications: Switching from p- to n-type behavior by additive addition. AIP Conf. Proc..

[11] Luo J., Krause B., Pötschke P. (2016). Melt-mixed thermoplastic composites containing carbon nanotubes for thermoelectric applications. AIMS Mater. Sci..

[12] Krause B., Barbier C., Levente J., Klaus M., Pötschke P. (2019). Screening of different carbon nanotubes in melt-mixed polymer composites with different polymer matrices for their thermoelectrical properties. J. Compos. Sci..

[13] Krause B., Konidakis I., Arjmand M., Sundararaj U., Fuge R., Liebscher M., Hampel S., Klaus M., Serpetzoglou E., Stratakis E. (2020). Nitrogen-doped carbon nanotube/polypropylene composites with negative Seebeck coefficient. J. Compos. Sci..

[14] Krause B., Bezugly V., Khavrus V., Ye L., Cuniberti G., Pötschke P. (2020). Boron doping of SWCNTs as way to enhance thermoelectric properties of melt mixed polypropylene/SWCNT composites. Energies.

[15] Tzounis L., Gärtner T., Liebscher M., Pötschke P., Stamm M., Voit B., Heinrich G. (2014). Influence of a cyclic butylene terephthalate oligomer on the processability and thermoelectric properties of polycarbonate/MWCNT nanocomposites. Polymer.

[16] Tzounis L., Liebscher M., Mäder E., Pötschke P., Stamm M., Logothetidis S. (2015). Thermal energy harvesting for large-scale applications using MWCNT-grafted glass fibers and polycarbonate-MWCNT nanocomposites. AIP Conf. Proc..

[17] Liebscher M., Gärtner T., Tzounis L., Mičušík M., Pötschke P., Stamm M., Heinrich G., Voit B. (2014). Influence of the MWCNT surface functionalization on the thermoelectric properties of melt-mixed polycarbonate composites. Compos. Sci. Technol..

[18] Gnanaseelan M., Chen Y., Luo J., Krause B., Pionteck J., Pötschke P., Qi H. (2018). Cellulose-carbon nanotube composite aerogels as novel thermoelectric materials. Compos. Sci. Technol..

[19] Paleo A.J., Vieira E.M.F., Wan K., Bondarchuk O., Cerqueira M.F., Goncalves L.M., Bilotti E., Alpuim P., Rocha A.M. (2019). Negative thermoelectric power of melt mixed vapor grown carbon nanofiber polypropylene composites. Carbon.

[20] Laird E.A., Kuemmeth F., Steele G.A., Grove-Rasmussen K., Nygård J., Flensberg K., Kouwenhoven L.P. (2015). Quantum transport in carbon nanotubes. Rev. Mod. Phys..

[21] Cho C., Culebras M., Wallace K.L., Song Y., Holder K., Hsu J., Yu C., Grunlan J.C. (2016). Stable n-type thermoelectric multilayer thin films with high power factor from carbonaceous nanofillers. Nano Energy.

[22] Ghorbanzadeh Ahangari M., Fereidoon A., Jahanshahi M., Ganji M.D. (2013). Electronic and mechanical properties of single-walled carbon nanotubes interacting with epoxy: A DFT study. Physica E.

[23] Fermi E. (1926). Zur Quantelung des einatomigen idealen Gases. Z. Phys..

[24] Torres L.E.F., Roche S., Charlier J.C. (2014). Introduction to Graphene-Based Nanomaterials: From Electronic Structure to Quantum Transport.

[25] Yang M., Koutsos V., Zaiser M. (2005). Interactions between polymers and carbon nanotubes: A molecular dynamics study. J. Phys. Chem. B.

[26] Plunkett A., Kröning K., Fiedler B. (2019). Highly optimized nitrogen-doped MWCNTs through in-depth parametric study using design of experiments. Nanomaterials.

[27] Jenschke W., Ullrich M., Krause B., Pötschke P. (2019). Messanlage zur Untersuchung des Seebeck effekts in polymermaterialien. Tech. Mess..

[28] Nonoguchi Y., Ohashi K., Kanazawa R., Ashiba K., Hata K., Nakagawa T., Adachi C., Tanase T., Kawai T. (2013). Systematic conversion of single walled carbon nanotubes into n-type thermoelectric materials by molecular dopants. Sci. Rep..

[29] Piao M., Na J., Choi J., Kim J., Kennedy G.P., Kim G., Roth S., Dettlaff-Weglikowska U. (2013). Increasing the thermoelectric power generated by composite film using chemically functionalized single-walled carbon nanotubes. Carbon.

[30] Kunadian I., Andrews R., Pinar Mengüc M., Qian D. (2009). Thermoelectric power generation using doped MWCNTs. Carbon.

[31] Fuge R., Liebscher M., Schröfl C., Oswald S., Leonhardt A., Mechtcherine V. (2016). Fragmentation characteristics of undoped and nitrogen-doped multiwalled carbon nanotubes in aqueous dispersion in dependence on the ultrasonication parameters. Diam. Relat. Mater..

